# Composite super-moiré lattices in double-aligned graphene heterostructures

**DOI:** 10.1126/sciadv.aay8897

**Published:** 2019-12-20

**Authors:** Zihao Wang, Yi Bo Wang, J. Yin, E. Tóvári, Y. Yang, L. Lin, M. Holwill, J. Birkbeck, D. J. Perello, Shuigang Xu, J. Zultak, R. V. Gorbachev, A. V. Kretinin, T. Taniguchi, K. Watanabe, S. V. Morozov, M. Anđelković, S. P. Milovanović, L. Covaci, F. M. Peeters, A. Mishchenko, A. K. Geim, K. S. Novoselov, Vladimir I. Fal’ko, Angelika Knothe, C. R. Woods

**Affiliations:** 1 Department of Physics and Astronomy, University of Manchester, Oxford Road, Manchester M13 9PL, UK.; 2 Institute of Nano Science, Nanjing University of Aeronautics and Astronautics, Nanjing 210016, China.; 3 National Graphene Institute, University of Manchester, Oxford Road, Manchester M13 9PL, UK.; 4 Henry Royce Institute for Advanced Materials, Oxford Road, Manchester M13 9PL, UK.; 5 Department of Materials, University of Manchester, Oxford Road, Manchester M13 9PL, UK.; 6 National Institute for Materials Science, 1-1 Namiki, Tsukuba 305-0044, Japan.; 7 Institute of Microelectronics Technology RAS, Chernogolovka 142432, Russia.; 8 Department of Physics, University of Antwerp, Groenenborgerlaan 171, Antwerp, Belgium.; 9 Centre for Advanced 2D Materials, National University of Singapore, Singapore 117546, Singapore.; 10 Chongqing 2D Materials Institute, Liangjiang New Area, Chongqing 400714, China.

## Abstract

When two-dimensional (2D) atomic crystals are brought into close proximity to form a van der Waals heterostructure, neighbouring crystals may influence each other’s properties. Of particular interest is when the two crystals closely match and a moiré pattern forms, resulting in modified electronic and excitonic spectra, crystal reconstruction, and more. Thus, moiré patterns are a viable tool for controlling the properties of 2D materials. However, the difference in periodicity of the two crystals limits the reconstruction and, thus, is a barrier to the low-energy regime. Here, we present a route to spectrum reconstruction at all energies. By using graphene which is aligned to two hexagonal boron nitride layers, one can make electrons scatter in the differential moiré pattern which results in spectral changes at arbitrarily low energies. Further, we demonstrate that the strength of this potential relies crucially on the atomic reconstruction of graphene within the differential moiré super cell.

## Teaser

Researchers combine moiré patterns to break the geometric limitations in graphene superlattices, and access new energy scales.

## INTRODUCTION

Van der Waals heterostructures allow combining different two-dimensional (2D) materials into functional stacks (*1*, *2*), which has already produced a range of interesting electronic (*3*, *4*) and optoelectronic (*5*–*8*) devices and resulted in observation of exciting physical phenomena. The large variety of the heterostructures is mainly due to the large selection of 2D materials. However, the assembly of van der Waals heterostructures allows one extra degree of freedom: Apart from the selection of the sequence of the 2D crystals, the individual crystals can be differently oriented with respect to each other. Previously, such control over the rotational alignment between crystals resulted in the observation of the resonant tunneling (*9*–*11*), renormalization of exciton binding energy (*12*), and insulating (*13*) and superconducting (*4*) states.

Probably one of the most spectacular results of the rotational alignment between different 2D crystals is the observation of the band reconstruction due to electron scattering on the moiré pattern in graphene aligned with hexagonal boron nitride (hBN). Because the lattice constants of graphene and hBN are relatively close to each other, the alignment between the two crystals leads to the formation of a moiré pattern (*14*, *15*) with a relatively small wave vector, which results in the appearance of the secondary Dirac points (*16*–*18*) in the electronic spectrum. Furthermore, the strong van der Waals interaction also leads to the atomic reconstruction of the graphene lattice (*19*–*22*). Unfortunately, the characteristic energies at which the electronic spectrum can be reconstructed are given by the difference between the lattice constants of graphene and hBN, which does not allow changes to be made to the low-energy part of the spectrum.

Here, we demonstrate how we can gain further control over the band reconstruction of graphene using the differential between two moiré patterns (super-moiré) created by top and bottom hBN in hBN/graphene/hBN heterostructures. Such super-moiré patterns are not related to the difference in the lattice constants between the two crystals and thus can be of any arbitrary wave number, which makes it possible to arrange the spectrum reconstruction at arbitrary low energies.

## RESULTS

**Fig. 1:**
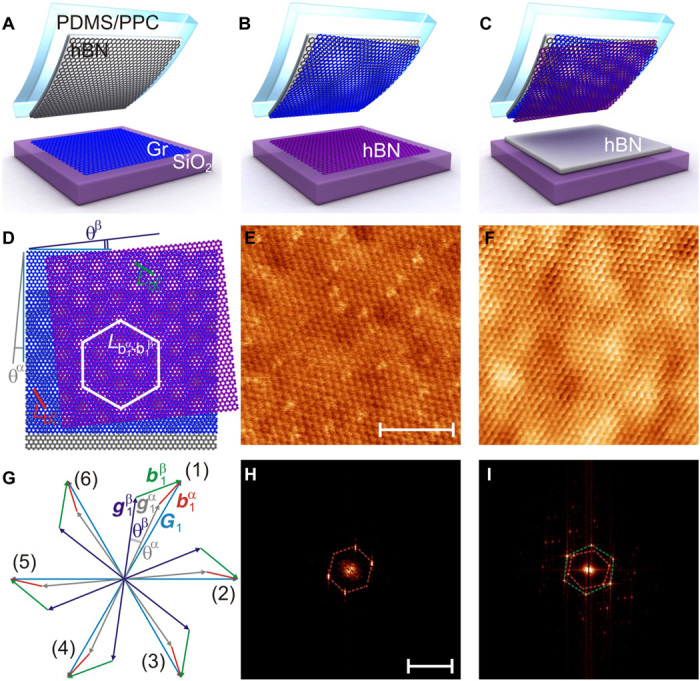
Device fabrication and characterization. (**A**) Step 1: A thick hBN layer is aligned and used to pick up graphene (Gr). (**B**) Step 2: A thin hBN layer is aligned and picked up forming a triple layer. (**C**) Step 3: The heterostructure is placed on top of a thick hBN layer at 15° rotation angle (the substrate). (**D**) Illustration of the two individual moiré patterns and super-moiré pattern for three overlapping hexagonal lattices. (**E**) AFM image of the moiré pattern after the graphene is picked up. This shows only one moiré periodicity. Scale bar, 100 nm [(F) shares this scale]. (**F**) AFM image of the moiré patterns after the second thin hBN layer is picked up. Here, periodicities due to both hBN layers are visible. (**G**) Reciprocal space image of graphene’s first Brillouin zone. ***G***_1_ (blue), $g_{1}^{\alpha }$ (gray), and $g_{1}^{\beta }$ (purple) are the reciprocal lattice vectors for graphene and α and β hBN layers, respectively. α and β are at angles θ^α^ and θ^β^ relative to graphene. $b_{1}^{\beta }$ (green) is the moiré between graphene and the β hBN layer. $b_{1}^{\alpha }$ (red) is the moiré between graphene and the α hBN layer. (**H**) Fourier transformation of the image in (E), displaying only one hexagonal periodic pattern (red dashed hexagon). Scale bar, 0.2 nm^−1^ [(I) shares this scale]. (**I**) Fourier transformation of the image in (F), showing two sets of distinct hexagonal patterns (red and green dashed hexagons).

To this end, we created encapsulated graphene devices where the graphene layer was aligned to both bottom and top hBN layers (alignment angles θ^α^ and θ^β^). The fabrication and transfer procedures have been previously described in (*23*) with the exception that not only the bottom but also the top hBN is now crystallographically aligned to the graphene. Briefly, we started by identifying the top hBN layer on SiO_2_. We then use a thin film of polypropylene carbonate (PPC) on Polydimethylsiloxane (PDMS) to lift the hBN from its substrate. This film then facilitates bringing the top hBN into contact with a graphene crystal (Fig. 1, A and B). We use very long and straight edges of the crystals to identify crystallographic axes and align them using a commercially available transfer rig (*24*). The graphene can then be lifted away from its substrate (Fig. 1B). At this point, we perform atomic force microscopy (AFM) (*14*, *15*, *19*) and Raman spectroscopy (*25*) experiments on the hBN/graphene bilayer to confirm the alignment. One such AFM image is presented in Fig. 1E, showing the characteristic hexagonal pattern (the Fourier transformation is shown in Fig. 1H). The crystals are then aligned and brought into contact with a second thin hBN layer (typically less than 1.5 nm or five atomic layers thick; Fig. 1B). This layer is also lifted away from its substrate, leaving a triple layer on the thin polymer film (Fig. 1C). We then perform AFM and Raman characterization again. Figure 1F and Fig. 1I are an example of one of our double-aligned moiré AFM images and its Fourier transformation for the case of the second hBN layer being one atomic layer thick, which allows one to see both moiré patterns simultaneously. Although not immediately clear in the real-space image, the Fourier transformation shows two sets of peaks corresponding to two hexagonal patterns (red and green dashed hexagons), as also schematically shown in Fig. 1D. The triple layer is then misaligned (~15°) and placed on top of a thick substrate hBN (Fig. 1C). Last, we use standard lithographic techniques to create the Hall bar geometry.

**Fig. 2:**
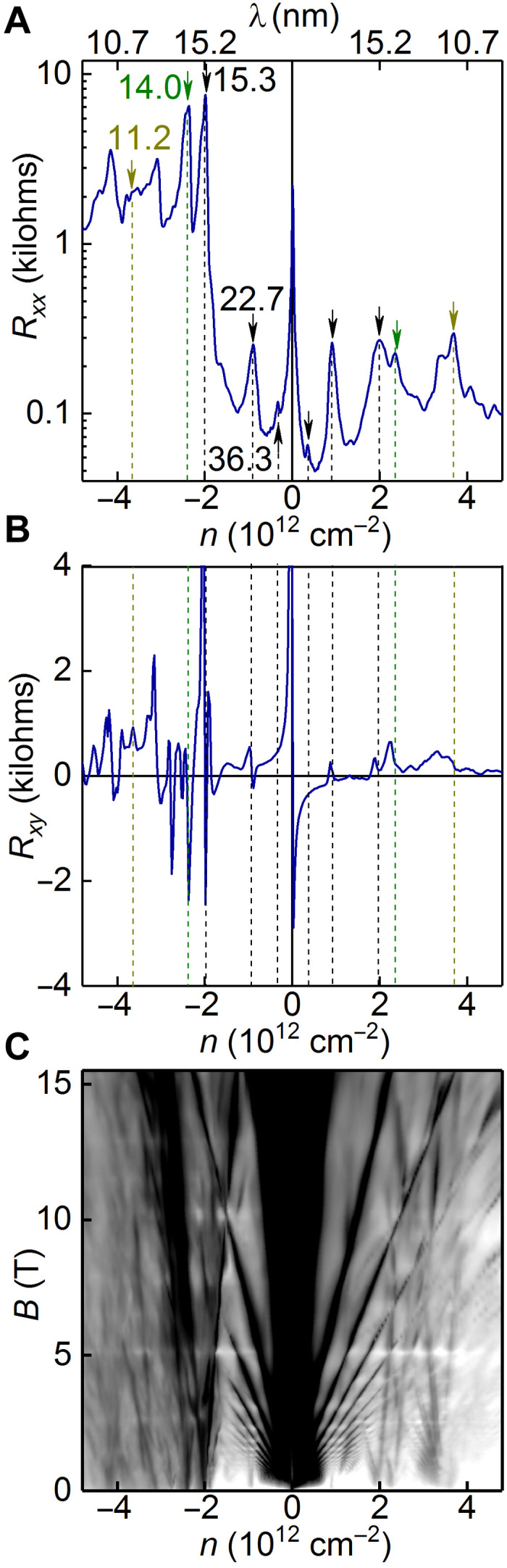
Transport properties of double-aligned hBN/graphene/hBN device. (**A**) *R_xx_* as a function of *n* for one of our devices with $b_{1}^{\alpha }$ ≈ 15.3 nm ($\varphi^{\alpha }$ = 0°), $b_{1}^{\beta }$≈ 14.0 nm ($\varphi^{\beta }$ = 0.4°). Lattice mismatch, δ, is taken as 1.64%. The moiré and super-moiré peaks are marked by arrows and also labeled with their periods on the hole side (in nanometers). The position of the peaks is symmetric with respect to holes and electrons. The top axis mark is labeled in the size of the moiré pattern that corresponds to the particular carrier concentration. Dashed lines correspond to the arrows and can be traced to those in (B). (**B**) *R_xy_* for the same device measured at *B* = 0.2 T, symmetrized to avoid contributions from *R_xx_*. The dashed lines can be traced to the arrows in (A) and correspond to the particular peaks in *R_xx_*. (**C**) Fan diagram σ*_xx_*(*n*, *B*) for the same device (scale black to white, 0.5 to 70 e^2^/hour). All measurements are performed at *T* = 1.7 K.

The longitudinal resistance (*R_xx_*) as a function of carrier concentration is shown in Fig. 2A. Here, apart from the resistance peak associated with the main Dirac point (*26*), several additional peaks can be seen. Most of such peaks correspond to the change of sign of the transversal (Hall) resistance (*R_xy_*) measured in nonquantized magnetic field (Fig. 2B). Typically, if graphene is aligned with only one hBN, then a single moiré pattern is produced, and only one secondary Dirac point for electrons and one for holes can be seen at concentrations that correspond to the wave vector determined by the periodicity of the moiré pattern (*14*–*18*). Aligning graphene to both the top and the bottom hBN will produce two moiré patterns (if θ^α^ and θ^β^ are not equivalent), which should result in two secondary Dirac points for electrons and two for holes. However, if electrons can feel potential from both moiré patterns simultaneously, then second-order processes can be allowed, which would result in the reconstruction of the electronic spectrum at many other wave vectors.

In quantized magnetic fields, Landau fans can be seen to originate from these peaks (Fig. 2C). The Landau fans for most peaks exhibit both positive and negative indices (positive and negative slopes in Fig. 2C), which suggests that those are originating from the additional Dirac points and not from the higher-order zone edges (*27*).

To interpret these additional peaks, we recall that the perfectly aligned graphene on hBN should produce moiré pattern with approximately 14-nm periodicity, which corresponds to carrier concentration *n* ≈ 2.3 × 10^12^ cm^−2^. Thus, for our double-aligned graphene, we interpret the most pronounced peaks at ±1.98 × 10^12^ and ±2.34 × 10^12^ cm^−2^ as coming from the moiré patterns from the top and bottom hBN layers.

The periodicity of a moiré pattern, *L*, can be related to the carrier concentration required to reach the edge of its first Brillouin zone by $n_{\text{SDP}}=\frac{8}{\sqrt{3}L^{2}}$. Using this, we get periodicities of 15.3 and 14.0 nm, respectively, for the two most prominent features. The moiré periodicities are dependent on both the lattice constant mismatch and the alignment angle, as given by $b^{\alpha ,\beta }=|b_{n}^{\alpha ,\beta }|=\frac{4\pi }{\sqrt{3}a}\sqrt{\delta^{2}+\theta^{\alpha ,\beta^{2}}}$, where $b_{n}^{\alpha ,\beta }=G_{n}-g_{n}^{\alpha ,\beta }$ (*n* = 1,…, 6) are the moiré reciprocal lattice vector between the α or β hBN layer (with reciprocal lattice vectors $g_{n}^{\alpha ,\beta }$) and graphene (with reciprocal lattice vectors ***G****_n_*), δ is the graphene-hBN lattice constant mismatch, θ^α, β^ is the misalignment angle for α or β, and *a* is graphene’s lattice constant. One of the observed periods is larger than that could be expected for graphene aligned with hBN [~15.3 nm; see (*14*–*19*)]. We attribute this slightly larger moiré period to stretching of graphene as it interacts more strongly with the two aligned hBN layers. Since the angle is zero, or sufficiently close to zero (δ ≫ θ), we may calculate a new δ. In this case, the lattice mismatch to achieve the periodicity of 15.3 nm should be ~1.64%. This corresponds to ~0.16% strain in the graphene crystal.

Then, we would like to notice the small peaks at ±0.35 × 10^12^ cm^−2^, which corresponds to the largest differential moiré pattern. From the carrier concentration, we can infer a periodicity of 35 nm. Further still, there is a pronounced peak at approximately ±0.90 × 10^12^ cm^−2^, which would yield a period of 22.7 nm. These features represent previously impossible periodicities for the graphene/hBN moiré pattern.

**Fig. 3:**
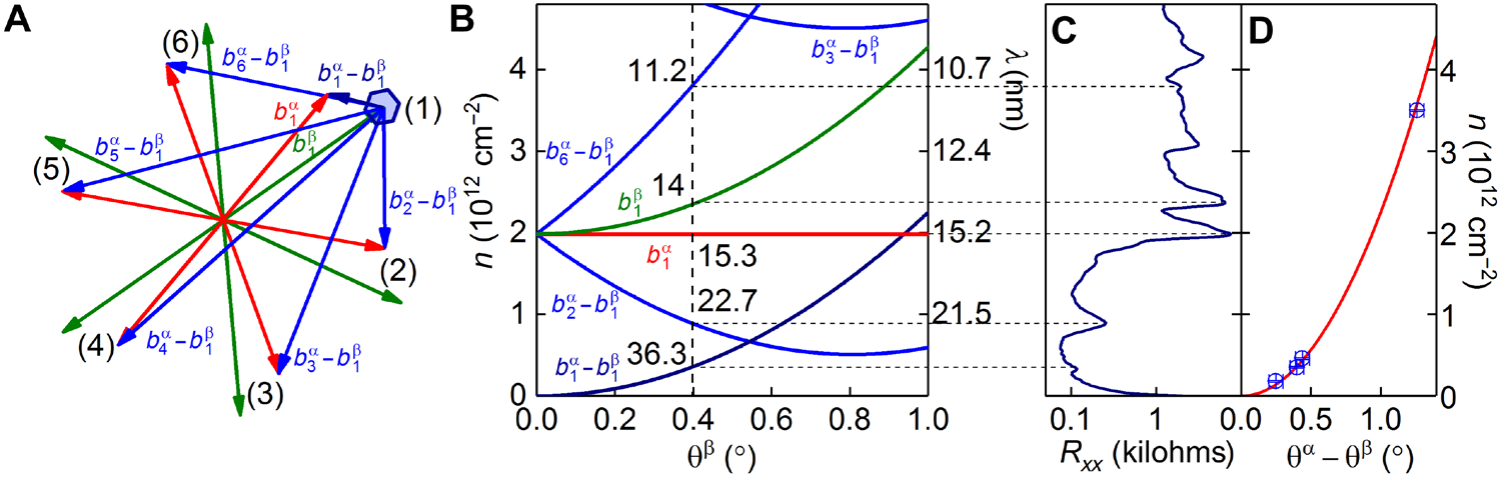
Super-moiré geometry. (**A**) Reciprocal-space image of the area around graphene’s *K* point. $b_{m}^{\alpha }$ (green) and $b_{m}^{\beta }$ (red) for *m* = 1, 2,…, 6 are the graphene-hBN moiré reciprocal lattice vectors. $b_{1}^{\alpha }-b_{m}^{\beta }$ (blue) are the six super-moiré reciprocal lattice vectors. The blue hexagonal area indicates the $b_{1}^{\alpha }-b_{1}^{\beta }$ first Brillouin zone. (**B**) Carrier concentration of the first Brillouin zone edge for the two moiré and four lowest-energy super-moiré features as a function of θ^β^(δ = 1.64%, θ^α^ = 0°). (**C**) *R_xx_* peak positions in carrier concentration. Dashed lines connect values of carrier concentration for θ^β^ = 0.4 in (B) to the position in (C). Each line matches a peak. (**D**) Carrier concentration of the $b_{1}^{\alpha }-b_{1}^{\beta }$ super-moiré feature versus ∣θ^α^ − θ^β^∣ for four of our samples (blue circles) and by calculation (red line).

In Fig. 3A, we schematically describe the geometric origin of the super-moiré features. $b_{m}^{\alpha }$ and $b_{k}^{\beta }$ (red and green vectors; *m* = 1, 2,…, 6, *k* = 1, 2,…, 6) are the α and β moiré patterns. Their combination produces six new super-moiré patterns by the combinations of the vectors. In Fig. 3A, we highlight the $b_{1}^{\alpha }-b_{k}^{\beta }$ vectors (blue). In Fig. 3B, we present the position of the moiré and super-moiré zone edges in carrier concentration as a function of the angle between the second hBN layer (θ^β^) and graphene for the case when the first hBN layer is held at zero angle mismatch (θ^α^ = 0) and δ= 1.64%, as calculated. For θ^β^ = 0.4°, the features correspond exactly to the observed peaks in *R_xx_* (as shown by the dashed lines connecting Fig. 3B and Fig. 3C) and sign reversal of *R_xy_*, thus revealing the presence of new secondary Dirac points in the low-energy electronic spectrum. Such low-energy peaks originate from the differential super-moirés, $b_{1}^{\alpha }-b_{1}^{\beta }$, $b_{1}^{\alpha }-b_{6}^{\beta }$, and $b_{1}^{\alpha }-b_{2}^{\beta }$. Further, in Fig. 3D, we show the position of the *R_xx_* peak in carrier concentration for the $b_{1}^{\alpha }-b_{2}^{\beta }$ super-moiré, against the calculated angle between the two hBNs (∣θ^α^ − θ^β^∣). This peak is unique because its period is independent of the graphene sheet as it is geometrically identical to moiré pattern between the two hBN layers. As expected, Fig. 3D shows that the peak position for four of our samples (blue circles) exactly follows expectation (red line).

**Fig. 4:**
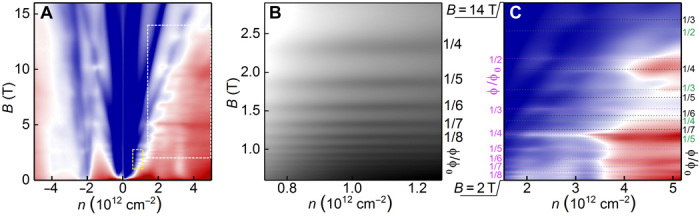
Brown-Zak oscillations in one of our double-aligned hBN/graphene/hBN devices. (**A**) Map of σ*_xx_*(*n*, *B*); scale of blue to red is 0.5 to 70 e^2^/hour. (**B**) Zoom-in view of the low-field part of the map, marked by the yellow dashed rectangle in (A); scale of black to white is 7 to 37 e^2^/hour. The Brown-Zak oscillations correspond to a moiré structure with a periodicity of 22.7 nm, and the fundamental field is 9.3 T. (**C**) Zoom-in view of the high-field part of the map, marked by the white dashed rectangle in (A); scale of blue to red is 7 to 37 e^2^/hour. The Brown-Zak oscillations that correspond to moiré structures of different periodicities are marked by dotted lines of different colors. Black, 15.3 nm (*B*_F_ = 20.5 T); green, 14.0 nm (*B*_F_ = 24.2 T); brown, 11.2 nm (*B*_F_ = 38 T). All measurements are performed at *T* = 70 K.

To check that all these peaks originate from the spectrum reconstruction because of scattering on the additional periodic potential, we measured the Brown-Zak oscillations at elevated temperatures (*T*) where cyclotron oscillations are suppressed (Fig. 4). At *T* > 70 K, oscillations independent of the carrier concentration can be clearly seen. At low fields *B* < 2.5 T (Fig. 4B), the oscillations are periodic in 1/*B* with the fundamental field *B*_f_ = 9.3 T. Assuming a hexagonal unit cell, such fundamental field can be calculated to correspond to a moiré periodicity of 22.7 nm, which corresponds to the peak in *R_xx_* at *n* = ±0.90 × 10^12^ cm^−2^ (Fig. 2A).

The behavior at high fields is more complex, as several Brown-Zak oscillations that originate from different periodicities overlap. However, by taking the periodicities, which correspond to the most prominent peaks in *R_xx_* at *B* = 0 (15.3, 14, and 11.2 nm; see Fig. 2A), we could identify most oscillations in terms of fractions of the flux quantum per the corresponding plaquette, labeled in Fig. 4C. Thus, the graphene-hBN moiré periods (14.0 and 15.3 nm) and super-moiré periods (11.2, 22.7, and 36.3 nm) each give features attributable to secondary Dirac points at well-understood values of carrier concentration. In addition, they produce clear Brown-Zak oscillations for unitary flux through moiré unit cells for the 11.2-, 14.0-, 15.3-, and 22.7-nm periods.

In addition to our previous observations, we would like to note that there are several unexplained features in *R_xx_* (Fig. 2A) and *R_xy_* (Fig. 2B), most pronounced at *n*_e_ ≈±3.2 × 10^12^ and ±4.1 × 10^12^ cm^−2^. One possible explanation for these features is higher-order moiré periodicities, that is, moiré patterns between super-moiré periods. However, the probability of such multiple scattering events diminishes strongly. Likewise, there could exist features due to super-moiré patterns between further zone edges (second, third,… Brillouin zone edges of the graphene/hBN moirés) or a more exotic superlattice (SL) phenomenon.

## DISCUSSION

Theoretically (*28*), moiré effects on graphene can be described in terms of a periodic SL potential applied to Dirac electrons produced by incommensurable lattices of two (top and bottom) hBN flakes

$$\hat{H}=vp•\sigma +\Sigma_{j=\pm }\Sigma_{n=0\ldots 5}[U_{0}^{j}+{\left(-1\right)}^{n}({\mathrm{iU}}_{3}^{j}\sigma_{3}+U_{1}^{j}\frac{a_{n}•\sigma }{a})]{\times e}^{ib_{n}^{j}•(r+j\frac{R}{2})}e^{iG_{n}•u(r,R)}$$ (1)

Here, σ_3_ and **σ** = (σ_1_, σ_2_) are Pauli matrices acting in the sublattice space of graphene’s Bloch states; *j* = ± identifies layers α (+) and β (−); $U_{0}^{j}$, $U_{1}^{j}$, and $U_{3}^{j}$ parameterize a smoothly varying moiré potential, the asymmetric sublattice on-site energies, and hopping between *A* and *B* sublattices, respectively [based on the earlier studies (*29*–*31*), $U_{0}^{j}\approx 8.5\;\text{meV}$, $U_{1}^{j}\approx -17\;\text{meV}$, and $U_{3}^{j}\approx -15\;\text{meV}$ for θ*^j^* ≪ δ]. Vector ***R*** describes the phase shift between moiré produced by hBN flakes α and β.

From this, the super-moiré periods for the individual moiré SLs (α, β) in graphene originate in two ways. One is due to the quantum mechanical interference, which appears in the second-order perturbation theory. In this case, Eq. 1 allows for the electron scattering from the combined *j* = ±SLs with the Bragg vectors $\;b_{m}^{\alpha }-b_{k}^{\beta }$. These are composed of different moiré SL reciprocal vectors, leading effectively to the SLs with Fourier components described in Figs. 1G and 3A. The second possibility is the reconstruction of graphene, which leads to a displacement field, ***u***(*r*, ***R***), generating mixing of the moiré SL’s reciprocal vectors. When this is considered, the longest-period super-moiré SL (*m* = 1, *k* = 1) that determines the low-energy part of graphene spectrum can be described by the following SL potential

$$\begin{array}{c} {\hat{H}}_{1,1}^{P\;}\approx -12U_{3}\omega_{\text{as}}\sigma_{3}-\Sigma_{m}[2U_{3}\omega_{\text{as}}\sigma_{3}+\frac{4U_{3}U_{1}}{\text{vb}}+i\frac{2U_{1}^{2}}{\text{vb}}\frac{b_{n}•\sigma }{b}]\times e^{ib_{m}^{\alpha }\;R}e^{i(b_{m}^{\alpha }-b_{m}^{\beta })•r}\\ {\hat{H}}_{1,1}^{\text{AP}\;}\approx \sum_{m}[i{\left(-1\right)}^{m}2U_{0}\omega_{\text{as}}-\frac{4U_{3}U_{1}}{\mathrm{vb}}+i\frac{2U_{1}^{2}}{\mathrm{vb}}\frac{b_{n}•\sigma }{b}]\times e^{ib_{m}^{\alpha }R}e^{i(b_{m}^{\alpha }-b_{m}^{\beta })•r} \end{array}$$ (2)

This expression was derived for θ^β^ ≪ δ for both parallel (P) and antiparallel (AP) mutual orientations of the two hBN crystals (hence, approximately $\;b_{m}^{\alpha }-b_{m}^{\beta }⊥b_{m}^{\alpha ,\beta }$), and, here, ω_as_ parameterizes the amplitude of inversion asymmetric component of strain.

Vital to this description is the understanding that the displacement field ***u***(*r*, ***R***) develops because of the competition between stacking-dependent van der Waals adhesion, of graphene and hBN, and elasticity of graphene. Previous work (*19*, *20*) has identified that the crystals form a 2D fixed-density commensurate state when graphene and hBN are close to perfect alignment. The effect relies upon strain and, thus, ***u***(*r*, ***R***), modulating with a period matching that of the moiré pattern to minimize adhesive and elastic energy. The commensurate state is characterized by hexagonal domains with increasingly sharp domain walls near θ = 0, observed in PeakForce AFM (*32*), and broadening of the 2D peak in the Raman spectrum (*19*, *25*).

**Fig. 5:**
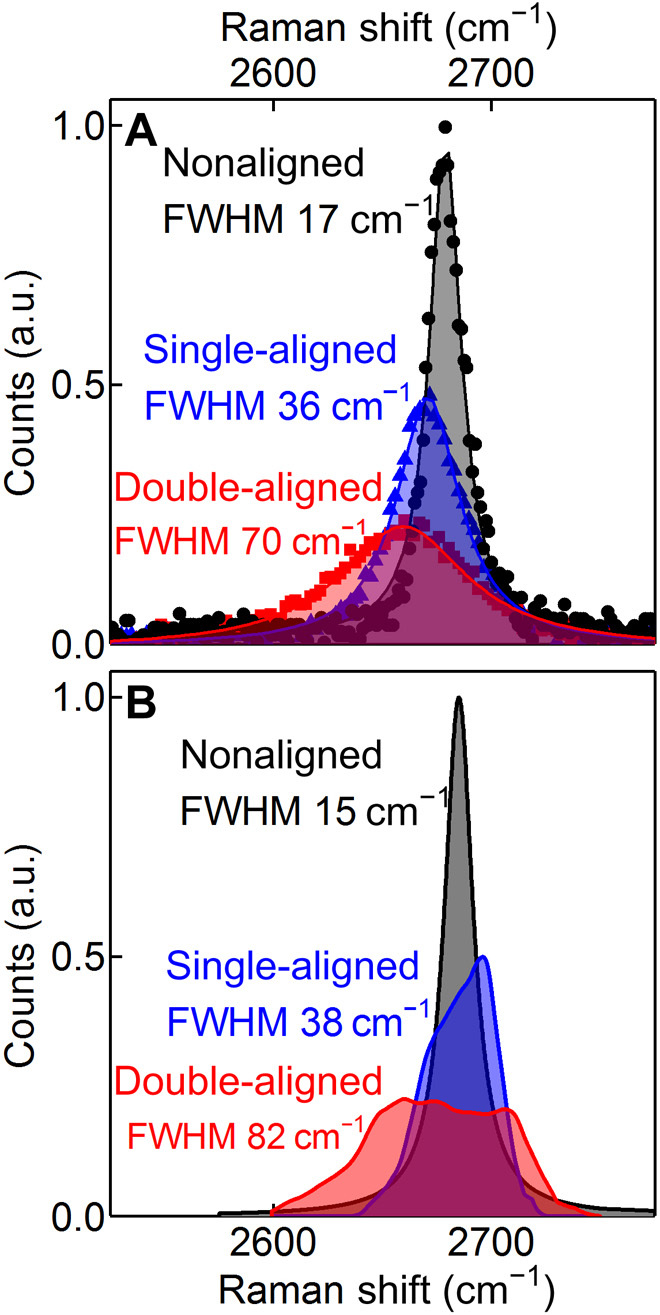
Strain distribution in the aligned graphene-hBN heterostructures. Raman spectra (2D peak region) for an unaligned sample (gray), single-aligned sample (blue), and double-aligned sample (red). (**A**) Experimental results. (**B**) Molecular dynamics relaxation simulations. a.u., arbitrary units.

To evaluate the degree of strain within our super-moiré samples, we have used Raman spectroscopy. In Fig. 5A, we show the 2D peak and its full width at half maximum (FWHM) for a typical unaligned sample (black circles), a sample aligned to one hBN (blue triangles), and one of our double-aligned samples (red squares). In the case that the two hBN layers may be treated entirely independently, the signature in the Raman spectrum would remain unchanged from the single-aligned case [*FWHM* (*2D*), ~36 cm^−1^]. However, it is clear that when a second aligned hBN layer is added, the width of the 2D peak increases by a factor of ~2 (Fig. 5A). We attribute this to restructuring of strain within the super-moiré unit cells. This observation supports the proposed model of the two moiré patterns mixing through strain fields. Therefore, ***u***(*r*, ***R***) should have periodicities described by $b_{m}^{\alpha }$ (α-moiré), $b_{k}^{\beta }$ (β-moiré), and $b_{m}^{\alpha }-b_{k}^{\beta }$ (super-moirés). This is also supported by molecular dynamics simulations of the relaxation in the double-aligned systems, as shown in Fig. 5B, where the Raman spectra of simulated relaxed configurations is presented (see the Supplementary Materials).

## CONCLUSION

To conclude, graphene’s electronic spectrum is substantially altered by scattering from super-moiré structures described by the preexisting moiré between graphene and its substrate and encapsulating hBN layers. These alterations may be considered in two ways: as double-scattering events from both graphene-hBN moiré patterns or as single-scattering events from a reconstructed graphene layer. Such super-moiré potential can be of an arbitrarily small wave vector (unlike moiré potential from single hBN aligned with graphene), which allows modification of the graphene band structure at arbitrarily low energies.

## MATERIALS AND METHODS

### Fabrication

In addition to the sample preparation described previously, the general process is as follows. We used PPC, or sometimes polymethyl methacrylate, spun on a thick PDMS membrane to facilitate moving and orientating the crystals. This membrane was used to pick up the first hBN layer. The crystal was then positioned and aligned to graphene before the two were brought into contact. We removed the membrane quickly which lifted the graphene off from its substrate. At this point, we inverted the membrane and perform various characterization techniques on the half-assembled heterostructure. We then picked up a further thin hBN crystal, before repeating our characterization. These previous steps could have been repeated multiple times to produce increasingly complex heterostructures with a variety of crystals (although, here, we limited ourselves to graphene and hBN). Last, the stack of crystals was positioned and brought into contact with a final “substrate” hBN. The membrane was removed slowly so that all the crystals were left on a SiO_2_ wafer.

### Atomic force microscopy

SL-resolution AFM images of the moiré patterns were taken in PeakForce Quantitative Nanomechanical Mapping (PF-QNM) mode on a Bruker Icon AFM. PF-QNM allows the capture of individual tip-sample force curves for each pixel in the image. These force curves were used to extract additional elastic information about the sample as well as topographic information. We used “ScanAsyst Fluid+” probes whose resolution is regularly better than 2 nm.

### Raman spectroscopy

Raman spectroscopy measurements were performed on a HORIBA XploRA PLUS Raman spectrometer. The laser wavelength was 532 nm with a power of 0.5 mW through a ×100 objective.

### Moiré Fourier analysis

The analysis of moiré patterns in double-aligned heterostructures was performed following an approach described in (*33*). The methodology was based on the Fourier analysis of moiré patterns appearing when two hexagonal lattices are combined.

### Molecular dynamics simulations

Molecular dynamics simulations were performed for the single-aligned hBN/graphene and the double-aligned hBN/graphene/hBN by allowing the relaxations of both hBN and graphene layers. We used the bond-order Brenner potentials for the graphene layer, Tersoff potentials for the B-N interaction in the hBN layers, and the Morse potential developed in (*34*) for the interlayer interactions. The simulations are performed within the “large-scale atomic/molecular massively parallel simulator” (*35*, *36*) by considering a disk of radius 120 nm. We fixed the atoms in a boundary region of 2 nm but allowed the relaxation of all other atoms. The total energy was minimized until the forces are below 10^−6^ eV/Å.

### Raman spectrum simulation

Using the relaxed structures, we calculated the shift of the 2D peak and the entire Raman spectrum of the graphene layer. Calculations were performed using the prescriptions given in (*37*–*39*).

## Supplementary Material

http://advances.sciencemag.org/cgi/content/full/5/12/eaay8897/DC1

Download PDF

Composite super-moiré lattices in double-aligned graphene heterostructures

## SUPPLEMENTARY MATERIALS

Supplementary material for this article is available at http://advances.sciencemag.org/cgi/content/full/5/12/eaay8897/DC1 More examples of double alignment Different fundamental frequencies of Brown-Zak oscillations Gap opening at the main Dirac point AFM of other double-aligned samples Uniformity in heterostructures Analysis of super-moiré peaks Tight-binding model Molecular dynamics simulations and Raman shift calculations Table S1. δ and θ^β^ for each device. Fig. S1. Transport properties of double-aligned encapsulated graphene devices. Fig. S2. Brown-Zak oscillations in sample 1. Fig. S3. Brown-Zak oscillations for sample 4. Fig. S4. Electron-hole symmetry in super-moiré features. Fig. S5. Gap opening in one of our double-aligned samples. Fig. S6. Examples of double-aligned samples. Fig. S7. Uniformity in double-aligned heterostructures. Fig. S8. Frequency analysis of different harmonics of hBN-graphene-hBN structure. Fig. S9. Super-moiré periods corresponding to different harmonics. Fig. S10. Molecular dynamics simulations of bond lengths in graphene-hBN superlattices. Reference (*40*)
