# Supplementary material for: Composite super-moiré lattices in double-aligned graphene heterostructures
Source: Sci Adv. 2019 Dec 20;5(12):eaay8897. doi: 10.1126/sciadv.aay8897 (PMC6989342; doi:10.1126/sciadv.aay8897)
Supplement: http://advances.sciencemag.org/cgi/content/full/5/12/eaay8897/DC1 [file supp_5_12_eaay8897__index.html]

Science Advances | Science AdvancesAAASSearchScience AdvancesMenu

## Supplementary Materials

**This PDF file includes:**

- More examples of double alignment
- Different fundamental frequencies of Brown-Zak oscillations
- Gap opening at the main Dirac point
- AFM of other double-aligned samples
- Uniformity in heterostructures
- Analysis of super-moiré peaks
- Tight-binding model
- Molecular dynamics simulations and Raman shift calculations
- Table S1. δ and θβ for each device.
- Fig. S1. Transport properties of double-aligned encapsulated graphene devices.
- Fig. S2. Brown-Zak oscillations in sample 1.
- Fig. S3. Brown-Zak oscillations for sample 4.
- Fig. S4. Electron-hole symmetry in super-moiré features.
- Fig. S5. Gap opening in one of our double-aligned samples.
- Fig. S6. Examples of double-aligned samples.
- Fig. S7. Uniformity in double-aligned heterostructures.
- Fig. S8. Frequency analysis of different harmonics of hBN-graphene-hBN structure.
- Fig. S9. Super-moiré periods corresponding to different harmonics.
- Fig. S10. Molecular dynamics simulations of bond lengths in graphene-hBN superlattices.
- Reference (*40*)

Download PDF

**Files in this Data Supplement:**

- Adobe PDF - aay8897\_SM.pdf
