# Supplementary material for: Composite super-moiré lattices in double-aligned graphene heterostructures
Source: Sci Adv. 2019 Dec 20;5(12):eaay8897. doi: 10.1126/sciadv.aay8897 (PMC6989342; doi:10.1126/sciadv.aay8897)
Supplement: Download PDF [file aay8897_SM.pdf]

Science Advances
AAAS

advances.sciencemag.org/cgi/content/full/5/12/eaay8897/DC1

Supplementary Materials for

Composite super-moiré lattices in double-aligned graphene heterostructures

Zihao Wang, Yi Bo Wang, J. Yin, E. Tóvári, Y. Yang, L. Lin, M. Holwill, J. Birkbeck, D. J. Perello, Shuigang Xu, J. Zultak, R. V. Gorbachev, A. V. Kretinin, T. Taniguchi, K. Watanabe, S. V. Morozov, M. Anđelković, S. P. Milovanović, L. Covaci, F. M. Peeters, A. Mishchenko, A. K. Geim, K. S. Novoselov, Vladimir I. Fal'ko, Angelika Knothe*, C. R. Woods*

*Corresponding author. Email: colin.woods@manchester.ac.uk (C.R.W.); angelika.knothe@manchester.ac.uk (A.K.)

Published 20 December 2019, *Sci. Adv.* **5**, eaay8897 (2019)
DOI: 10.1126/sciadv.aay8897

This PDF file includes:

More examples of double alignment
Different fundamental frequencies of Brown-Zak oscillations
Gap opening at the main Dirac point
AFM of other double-aligned samples
Uniformity in heterostructures
Analysis of super-moiré peaks
Tight-binding model
Molecular dynamics simulations and Raman shift calculations
Table S1. δ and θ^{β} for each device.
Fig. S1. Transport properties of double-aligned encapsulated graphene devices.
Fig. S2. Brown-Zak oscillations in sample 1.
Fig. S3. Brown-Zak oscillations for sample 4.
Fig. S4. Electron-hole symmetry in super-moiré features.
Fig. S5. Gap opening in one of our double-aligned samples.
Fig. S6. Examples of double-aligned samples.
Fig. S7. Uniformity in double-aligned heterostructures.
Fig. S8. Frequency analysis of different harmonics of hBN-graphene-hBN structure.
Fig. S9. Super-moiré periods corresponding to different harmonics.
Fig. S10. Molecular dynamics simulations of bond lengths in graphene-hBN superlattices.
Reference (*40*)

Supplementary Information

Contents:

Details of the super-moiré superlattice perturbation theory

Details of the super-moiré superlattice perturbation theory

We study the long-range periodic "super"-moire pattern which appears due to beatings between the two moires at the top and bottom interfaces. The six reciprocal lattice vectors of the top (bottom) moire pattern are given by $\mathbf{b}_m^\alpha = \mathbf{G}_m - \mathbf{g}_m^\alpha$ ($\mathbf{b}_m^\beta = \mathbf{G}_m - \mathbf{g}_m^\beta$) for $m = 0, \ldots, 5$, where $\mathbf{G}_m$ denote the reciprocal lattice vectors of graphene, and $\mathbf{g}_m^\alpha$ ($\mathbf{g}_m^\beta$) are the reciprocal lattice vectors of the top (bottom) hBN. From these, we construct the combinations $\mathbf{d}_{m,k} = \mathbf{b}_m^\alpha - \mathbf{b}_k^\beta$. For twist different angles θ^α (θ^β) of the top (bottom) hBN layer, these become very small or vanish completely and hence constitute the shortest reciprocal lattice vectors of the "super"-moire pattern. These cases are studied below.

Derivation of the Hamiltonians of shortest period

The low-energy contribution (for the shortest effective Bragg vectors $\mathbf{d}_{m,k} = \mathbf{b}^\alpha{}_m - \mathbf{b}_k^\beta$) of the superlattice Hamiltonian which originates from interference reads in second order perturbation theory

$$\mathrm{H}_{n,m}^{int} = \delta\mathrm{H}_{\alpha\beta}^{(2)} + \delta\mathrm{H}_{\beta\alpha}^{(2)}$$

with

$$\delta \mathrm{H}^{(2)}_{\substack{\alpha\beta\\(\beta\alpha)}} = \sum_{m,k} \frac{v}{v^2 b^2} \Bigg(\Bigg[U_0 U_1 (-1)^k \frac{1}{a} \boldsymbol{b}_k^{\beta(\alpha)} . \boldsymbol{a}_k + U_0 U_1 (-1)^m \frac{1}{a} \boldsymbol{b}_k^{\beta(\alpha)} . \boldsymbol{a}_m$$

$$+ U_1 U_3 (-1)^{m+k} \frac{1}{a} (\boldsymbol{\ell}_z \times \boldsymbol{b}_k^{\beta(\alpha)}).(\boldsymbol{a}_k + \boldsymbol{a}_m) \Bigg] e^{\mp i \frac{\mathbf{R}}{2}(\boldsymbol{b}_k^\beta + \boldsymbol{b}_m^\alpha)} \ e^{i(\boldsymbol{b}_k^\beta - \boldsymbol{b}_m^\alpha).\mathbf{r}}$$

$$+ i \frac{1}{a} \Bigg[(-1)^k U_1 U_0 (\boldsymbol{\ell}_z \times \boldsymbol{b}_k^{\beta(\alpha)}).\boldsymbol{a}_k - (-1)^m U_1 U_0 (\boldsymbol{\ell}_z \times \boldsymbol{b}_k^{\beta(\alpha)}).\boldsymbol{a}_m$$

$$- U_1 U_3 (-1)^{m+k} \boldsymbol{b}_k^{\beta(\alpha)}.(\boldsymbol{a}_k - \boldsymbol{a}_m) \Bigg] \sigma_3 \, e^{\mp i \frac{\mathbf{R}}{2}(\boldsymbol{b}_k^\beta + \boldsymbol{b}_m^\alpha)} \ e^{i(\boldsymbol{b}_k^\beta - \boldsymbol{b}_m^\alpha).\mathbf{r}}$$

$$+ \Bigg[i(-1)^{m+k} U_1^2 \frac{1}{a^2} \big[(\boldsymbol{a}_m.\boldsymbol{b}_k^{\beta(\alpha)}).(\boldsymbol{a}_k.\boldsymbol{\sigma}) + [\boldsymbol{a}_m.(\boldsymbol{\ell}_z \times \boldsymbol{b}_k^{\beta(\alpha)})].[(\boldsymbol{\ell}_z \times \boldsymbol{a}_k).\boldsymbol{\sigma}] \big] \Bigg] e^{\mp i \frac{\mathbf{R}}{2}(\boldsymbol{b}_{n'}^\beta + \boldsymbol{b}_m^\alpha)} \ e^{i(\boldsymbol{b}_k^\beta - \boldsymbol{b}_m^\alpha).\mathbf{r}}$$

$$+ \Bigg[U_0^2 \boldsymbol{b}_k^{\beta(\alpha)} - (-1)^{m+k} U_3^2 \boldsymbol{b}_k^{\beta(\alpha)} + U_0 U_3 \big((-1)^k + (-1)^m \big) (\boldsymbol{\ell}_z \times \boldsymbol{b}_{n'}^{\beta(\alpha)}) \Bigg] \boldsymbol{\sigma} e^{\mp i \frac{\mathbf{R}}{2}(\boldsymbol{b}_k^\beta + \boldsymbol{b}_m^\alpha)} \ e^{i(\boldsymbol{b}_k^\beta - \boldsymbol{b}_m^\alpha).\mathbf{r}} \Bigg) \tag{S1}$$

while the corresponding contribution due to strain caused by reconstruction is given by (40)

$$\mathrm{H}^{rec} = \sum_{m,k} \Bigg(i \Big[U_0^\beta \mathbf{G}_m \cdot \mathbf{u}_{\mathbf{b}_k^\alpha} - U_0^\alpha \mathbf{G}_m \cdot \mathbf{u}_{-\mathbf{b}_k^\beta} \Big] + i\sigma_3 \Big[(-1)^m i U_3^\beta \mathbf{G}_m \cdot \mathbf{u}_{\mathbf{b}_k^\alpha} + (-1)^k i U_3^\alpha \mathbf{G}_m \cdot \mathbf{u}_{-\mathbf{b}_k^\beta} \Big]$$

$$+ \Big[(-1)^m i U_1^\beta \frac{\mathbf{a}_m}{a} \boldsymbol{\sigma} \mathbf{G}_m \cdot \mathbf{u}_{\mathbf{b}_k^\alpha} - (-1)^k i U_1^\alpha \frac{\mathbf{a}_k}{a} \boldsymbol{\sigma} \mathbf{G}_m \cdot \mathbf{u}_{-\mathbf{b}_k^\beta} \Big] \Bigg) e^{i(\mathbf{b}_m^\alpha + \mathbf{b}_k^\beta)\cdot \frac{\mathbf{R}}{2}} \ e^{i \mathbf{d}_{mk} \cdot \mathbf{r}} \tag{S2}$$

We consider the terms of the Hamiltonians above with $m = k$, under the assumption of very small angles (for which $U_i^\beta \approx U_i^\alpha =: U_i$)

$$\delta \mathrm{H}^{int}_{mm} = \delta \mathrm{H}^{(2)}_{\alpha\beta} + \delta \mathrm{H}^{(2)}_{\beta\alpha}$$

$$= \sum_m \frac{v}{v^2 b^2} \Bigg(\Bigg[2 U_0 U_1 (-1)^m \frac{1}{a} (\boldsymbol{b}_m^\alpha - \boldsymbol{b}_m^\beta).\boldsymbol{a}_m$$

$$+ 2 U_1 U_3 \frac{1}{a} \big[(\boldsymbol{\ell}_z \times \boldsymbol{b}_m^\alpha).\boldsymbol{a}_m + (\boldsymbol{\ell}_z \times \boldsymbol{b}_m^\beta).\boldsymbol{a}_m \big] \Bigg] e^{-i \frac{\mathbf{R}}{2}(\boldsymbol{b}_m^\alpha + \boldsymbol{b}_m^\beta)} \ e^{i(\boldsymbol{b}_m^\alpha - \boldsymbol{b}_m^\beta).\mathbf{r}}$$

$$+ \Bigg[i U_1^2 \frac{1}{a^2} \Big[[(\boldsymbol{a}_m.\boldsymbol{b}_m^\alpha) - (\boldsymbol{a}_m.\boldsymbol{b}_m^\beta)].(\boldsymbol{a}_m.\boldsymbol{\sigma})$$

$$+ \Big([\boldsymbol{a}_m.(\boldsymbol{\ell}_z \times \boldsymbol{b}_m^\beta)] + [\boldsymbol{a}_m.(\boldsymbol{\ell}_z \times \boldsymbol{b}_m^\alpha)] \Big).[(\boldsymbol{\ell}_z \times \boldsymbol{a}_m).\boldsymbol{\sigma}] \Big] \Bigg] e^{-i \frac{\mathbf{R}}{2}(\boldsymbol{b}_m^\alpha + \boldsymbol{b}_m^\beta)} \ e^{i(\boldsymbol{b}_m^\alpha - \boldsymbol{b}_m^\beta).\mathbf{r}}$$

$$+ \Bigg[U_0^2 (\boldsymbol{b}_n^\alpha - \boldsymbol{b}_m^\beta) - U_3^2 (\boldsymbol{b}_m^\alpha - \boldsymbol{b}_m^\beta) + 2 U_0 U_3 (-1)^m [(\boldsymbol{\ell}_z \times \boldsymbol{b}_m^\beta) + (\boldsymbol{\ell}_z \times \boldsymbol{b}_m^\alpha)] \Bigg] \boldsymbol{\sigma} e^{-i \frac{\mathbf{R}}{2}(\boldsymbol{b}_m^\alpha + \boldsymbol{b}_m^\beta)} \ e^{i(\boldsymbol{b}_m^\alpha - \boldsymbol{b}_m^\beta).\mathbf{r}} \Bigg) \tag{S3}$$

For the contribution due to strain, the cases in which the two hBN layers are either parallel, or antiparallel with respect to each other must be distinguished:

Parallel case

$$
\begin{aligned}
\mathrm{H}^{P}_{m,m} = &- U_0(w_s^\alpha + w_s^\beta) f_1^{(m,m)}(\mathbf{r}) - U_3(w_s^\alpha - w_s^\beta) f_2^{(m,m)}(\mathbf{r})\sigma_3 \\
&+ U_1(w_s^\alpha + w_s^\beta)\frac{\sqrt{3}a}{4\pi}\frac{1}{(\theta^\beta - \theta')}\boldsymbol{\nabla} f_2^{(m,m)}(\mathbf{r})\boldsymbol{\sigma} \\
&+ U_0(w_{as}^\alpha - w_{as}^\beta) f_2^{(m,m)}(\mathbf{r}) - U_3(w_{as}^\alpha + w_{as}^\beta) f_1^{(m,m)}(\mathbf{r})\sigma_3 \\
&+ U_1(w_{as}^\alpha - w_{as}^\beta)\frac{\sqrt{3}a}{4\pi}\frac{1}{(\theta^\beta - \theta^\alpha)}\boldsymbol{\nabla} f_1^{(m,m)}(\mathbf{r})\boldsymbol{\sigma}
\end{aligned}
\tag{S4}
$$

Antiparallel case

$$
\begin{aligned}
\mathrm{H}^{AP}_{m,m} = &- U_0(w_s^\alpha - w_s^\beta) f_1^{(m,m)}(\mathbf{r}) - U_3(w_s^\alpha + w_s^\beta) f_2^{(m,m)}(\mathbf{r})\sigma_3 \\
&+ U_1(w_s^\alpha - w_s^\beta)\frac{\sqrt{3}a}{4\pi}\frac{1}{(\theta^\beta - \theta^\alpha)}\boldsymbol{\nabla} f_2^{(m,m)}(\mathbf{r})\boldsymbol{\sigma} \\
&+ U_0(w_{as}^\alpha + w_{as}^\beta) f_2^{(m,m)}(\mathbf{r}) - U_3(w_{as}^\alpha - w_{as}^\beta) f_1^{(m,m)}(\mathbf{r})\sigma_3 \\
&+ U_1(w_{as}^\alpha + w_{as}^\beta)\frac{\sqrt{3}a}{4\pi}\frac{1}{(\theta^\beta - \theta^\alpha)}\boldsymbol{\nabla} f_1^{(m,m)}(\mathbf{r})\boldsymbol{\sigma}
\end{aligned}
\tag{S5}
$$

in terms of the functions $f_1^{(m,m)}(\mathbf{r}) = \sum_m e^{i(\mathbf{b}_m^\alpha + \mathbf{b}_m^\beta)\cdot\frac{\mathbf{R}}{2}} e^{i\mathbf{d}_{m,m}\cdot\mathbf{r}}$, $f_2^{(m,m)}(\mathbf{r}) = i\sum_m (-1)^m e^{i(\mathbf{b}_m^\alpha + \mathbf{b}_m^\beta)\cdot\frac{\mathbf{R}}{2}} e^{i\mathbf{d}_{m,m}\cdot\mathbf{r}}$ and $\Re[\mathbf{G}_m \cdot \mathbf{u}_{\mathbf{b}_m^\alpha}] = \Re[\mathbf{G}_m \cdot \mathbf{u}_{-\mathbf{b}_m^\alpha}] = -(-1)^n w_{as}^\alpha$, $\Im[\mathbf{G}_m \cdot \mathbf{u}_{\mathbf{b}_m^\alpha}] = -\Im[\mathbf{G}_m \cdot \mathbf{u}_{-\mathbf{b}_m^\alpha}] = w_s^\alpha$.

Under the assumption that for small and almost equal angles $w_i^\alpha \approx w_i^\beta \approx w_i$, and keeping in mind that the gradient terms in equations (S3), (S4), (S5) can be removed by a gauge transformation (28) we arrive at the superlattice Hamiltonians $\mathrm{H}_{m,m}$ of equation (2) in the main text.

All combinations of short effective moire Bragg vectors

For all the possible shortest effective Bragg vectors $\mathbf{d}_{m,k}$ we list the functions that describe the period and density of the corresponding super-moire

structures

$$\bigstar\ \mathbf{d}_{m,m} = \mathbf{b}^{\alpha}_{m} - \mathbf{b}^{\beta}_{m} = \frac{4\pi}{\sqrt{3}\mathrm{a}(1+\delta)} \begin{pmatrix} \sin\theta^{\alpha} - \sin\theta^{\beta} \\ \cos\theta^{\beta} - \cos\theta^{\alpha} \end{pmatrix}$$

$$A_{m,m} = \mathrm{a}(\delta+1)\frac{1}{\sqrt{2-2\cos(\theta^{\alpha}-\theta^{\beta})}}$$

$$n_{m,m} = -\frac{16[\cos(\theta^{\alpha}-\theta^{\beta})-1]}{\sqrt{3}\mathrm{a}^2(\delta+1)^2}$$

Divergence of $A_{m,m}$ and zero $n_{m,m}$ for all twist angles with $\theta^{\alpha} = \theta^{\beta}$

$$\bigstar\ \mathbf{d}_{m,m+1} = \mathbf{b}^{\alpha}_{m} - \mathbf{b}^{\beta}_{m+1} = \frac{2\pi}{\mathrm{a}(1+\delta)} \begin{pmatrix} \frac{3\delta+2\sqrt{3}\sin\theta^{\alpha}-\sqrt{3}\sin\theta^{\beta}-3\cos\theta^{\beta}+3}{3} \\ \frac{\delta-2\cos\theta^{\alpha}-\sqrt{3}\sin\theta^{\beta}+\cos\theta^{\beta}+1}{\sqrt{3}} \end{pmatrix}$$

$$\begin{aligned} &A_{m,m+1} \\ =&\frac{2\sqrt{3}\mathrm{a}(\delta+1)}{3\left(\delta-2\cos\theta^{\alpha}-\sqrt{3}\sin\theta^{\beta}+\cos\theta^{\beta}+1\right)^2+\left(3\delta+2\sqrt{3}\sin\theta^{\alpha}-\sqrt{3}\sin\theta^{\beta}-3\cos\theta^{\beta}+3\right)^2} \\ \approx&\frac{\mathrm{a}(\delta+1)}{\delta^2+\sqrt{3}\delta(\theta^{\alpha}-\theta^{\beta})+(\theta^{\alpha})^2-\theta^{\alpha}\theta^{\beta}+(\theta^{\beta})^2} \end{aligned}$$

$$\begin{aligned} &n_{m,m+1} \\ =&\frac{2\left(3\left(\delta-2\cos\theta^{\alpha}-\sqrt{3}\sin\theta^{\beta}+\cos\theta^{\beta}+1\right)^2+\left(3\delta+2\sqrt{3}\sin\theta^{\alpha}-\sqrt{3}\sin\theta^{\beta}-3\cos\theta^{\beta}+3\right)^2\right)}{3\sqrt{3}\mathrm{a}^2(\delta+1)^2} \\ \approx&\frac{8\left(\delta^2-\sqrt{3}\delta(\theta^{\beta}-\theta^{\alpha})+(\theta^{\alpha})^2-\theta^{\alpha}\theta^{\beta}+(\theta^{\beta})^2\right)}{\sqrt{3}\mathrm{a}^2(\delta+1)^2} \end{aligned}$$

Angles of the critical points (Divergence of A and zero n)

$$\theta^{\alpha} = -\theta^{\beta} = \tan^{-1}\left[\frac{\sqrt{-(\delta-1)(\delta+3)}\delta+\sqrt{-(\delta-1)(\delta+3)}-\sqrt{3}}{\delta(\delta+2)-2}\right] \approx -\frac{\delta}{\sqrt{3}}$$

$$\bigstar\ \mathbf{d}_{m,m+2} = \mathbf{b}^{\alpha}_{m} - \mathbf{b}^{\beta}_{m+2} = \frac{2\pi}{\mathrm{a}(1+\delta)} \begin{pmatrix} \frac{3\delta+2\sqrt{3}\sin\theta^{\alpha}+\sqrt{3}\sin\theta^{\beta}-3\theta^{\beta}+3}{3} \\ \frac{-3\delta+2\cos\theta^{\alpha}+\sqrt{3}\sin\theta^{\beta}+\cos\theta^{\beta}-3}{\sqrt{3}} \end{pmatrix}$$

$$\begin{aligned} &A_{m,m+2} = \\ &\frac{2\sqrt{3}(\delta+1)\,\mathrm{a}}{\sqrt{3\left(-3\delta+2\cos\theta^{\alpha}+\sqrt{3}\sin\theta^{\beta}+\cos\theta^{\beta}-3\right)^2+\left(3\delta+2\sqrt{3}\sin\theta^{\alpha}+\sqrt{3}\sin\theta^{\beta}-3\cos\theta^{\beta}+3\right)^2}} \\ \approx&\frac{\mathrm{a}(\delta+1)}{\sqrt{3\delta^2+\sqrt{3}\delta(\theta^{\alpha}-\theta^{\beta})+(\theta^{\alpha})^2+\theta^{\alpha}\theta^{\beta}+\theta^{\beta\,2}}} \end{aligned}$$

$$n_{m,m+2}$$
$$= \frac{2\left(3\left(-3\delta+2\cos\theta^\alpha+\sqrt{3}\sin\theta^\beta+\cos\theta^\beta-3\right)^2+\left(3\delta+2\sqrt{3}\sin\theta^\alpha+\sqrt{3}\sin\theta^\beta-3\cos\theta^\beta+3\right)^2\right)}{3\sqrt{3}\mathrm{a}^2(\delta+1)^2}$$
$$\approx \frac{8\left(3\delta^2+\sqrt{3}\delta(\theta^\alpha-\theta^\beta)+\theta^2+\theta^\alpha\theta^\beta+(\theta^\beta)^2\right)}{\sqrt{3}\mathrm{a}^2(\delta+1)^2}$$

Angles of the critical points (Divergence of A and zero n)

$$\theta^\alpha=-\theta^\beta=\tan^{-1}\left[\frac{\sqrt{3}\left(-\delta+\sqrt{1-3\delta(\delta+2)}-1\right)}{3\delta+\sqrt{1-3\delta(\delta+2)}+3}\right]\approx-\sqrt{3}\delta$$

★ $\mathbf{d}_{m,m+3}=\mathbf{b}^\alpha_m-\mathbf{b}^\beta_{m+3}=\frac{4\pi}{\sqrt{3}\mathrm{a}}\begin{pmatrix}\frac{\sin\theta^\alpha+\sin\theta^\beta}{1+\delta}\\ 2-\frac{\cos\theta^\alpha+\cos\theta^\beta}{\delta+1}\end{pmatrix}$

$$A_{m,m+3}$$
$$=\mathrm{a}\frac{(\delta+1)}{\sqrt{2}}\frac{1}{\sqrt{-2(\delta+1)\cos\theta^\alpha-2(\delta+1)\cos\theta^\beta+2\delta(\delta+2)+\cos(\theta^\alpha-\theta^\beta)+3}}$$

$$n_{m,m+3}$$
$$=-\frac{16[-2(\delta+1)\cos\theta^\alpha-2(\delta+1)\cos\theta^\beta+2\delta(\delta+2)+\cos(\theta^\alpha-\theta^\beta)+3]}{\sqrt{3}\mathrm{a}^2(\delta+1)^2}$$

with maximum of $A_{m,m+3}$ and minimum of $n_{m,m+3}$ at $\theta^\alpha=\theta^\beta=0$

★ $\mathbf{d}_{m,m+4}=\mathbf{b}^\alpha_m-\mathbf{b}^\beta_{m+4}=\frac{2\pi}{\mathrm{a}(1+\delta)}\begin{pmatrix}\frac{-3\delta+2\sqrt{3}\sin\theta+\sqrt{3}\sin\theta'+3\cos\theta'-3}{3}\\ \frac{3\delta-2\cos\theta+\sqrt{3}\sin\theta'-\cos\theta'+3}{\sqrt{3}}\end{pmatrix}$

$$A_{m,m+4}$$
$$=\frac{2\sqrt{3}(\delta+1)\ \mathrm{a}}{\sqrt{3\left(3\delta-2\cos\theta^\alpha+\sqrt{3}\sin\theta^\beta-\cos\theta^\beta+3\right)^2+\left(-3\delta+2\sqrt{3}\sin\theta^\alpha+\sqrt{3}\sin\theta^\beta+3\cos\theta^\beta-3\right)^2}}$$
$$\approx\frac{\mathrm{a}(\delta+1)}{\sqrt{3\delta^2+\sqrt{3}\delta(\theta^\beta-\theta^\alpha)+(\theta^\alpha)^2+\theta^\alpha\theta^\beta+\theta^{\beta\,2}}}$$

$$n_{m,m+4}$$
$$=\frac{2\left(3\left(3\delta-2\cos\theta^\alpha+\sqrt{3}\sin\theta^\beta-\cos\theta^\beta+3\right)^2+\left(-3\delta+2\sqrt{3}\sin\theta^\alpha+\sqrt{3}\sin\theta^\beta+3\cos\theta^\beta-3\right)^2\right)}{3\sqrt{3}\mathrm{a}^2(\delta+1)^2}$$
$$\approx\frac{8\left(3\delta^2+\sqrt{3}\delta(\theta^\beta-\theta^\alpha)+(\theta^\alpha)^2+\theta^\alpha\theta^\beta+(\theta^\beta)^2\right)}{\sqrt{3}\mathrm{a}^2(\delta+1)^2}$$

Angles of the critical points (Divergence of A and zero n)

$$\theta^{\alpha} = -\theta^{\beta} = \tan^{-1}\left[\frac{\sqrt{3}\left(\delta - \sqrt{1-3\delta(\delta+2)}+1\right)}{3\delta+\sqrt{1-3\delta(\delta+2)}+3}\right] \approx \sqrt{3}\delta$$

$$\bigstar\ \mathbf{d}_{m,m+5} = \mathbf{b}^{\alpha}_{m} - \mathbf{b}^{\beta}_{m+5} = \tfrac{2\pi}{\mathrm{a}(1+\delta)}\begin{pmatrix}\frac{3\delta-2\sqrt{3}\sin\theta^{\alpha}+\sqrt{3}\sin\theta^{\beta}-3\cos\theta^{\beta}+3}{3}\\ \frac{\delta-2\cos\theta^{\alpha}+\sqrt{3}\sin\theta^{\beta}+\cos\theta^{\beta}+1}{\sqrt{3}}\end{pmatrix}$$

$$\begin{aligned}&A_{m,m+5}\\ =&\frac{2\sqrt{3}\mathrm{a}(\delta+1)}{\sqrt{3\left(\delta-2\cos\theta^{\alpha}+\sqrt{3}\sin\theta^{\beta}+\cos\theta^{\beta}+1\right)^2+\left(3\delta-2\sqrt{3}\sin\theta^{\alpha}+\sqrt{3}\sin\theta^{\beta}-3\cos\theta^{\beta}+3\right)^2}}\\ \approx&\mathrm{a}\frac{\delta+1}{\sqrt{\delta^2+\sqrt{3}\delta(\theta^{\beta}-\theta^{\alpha})+(\theta^{\alpha})^2-\theta^{\alpha}\theta^{\beta}+(\theta^{\beta})^2}}\end{aligned}$$

$$\begin{aligned}&n_{m,m+5}\\ =&\frac{2\left(3\left(\delta-2\cos\theta^{\alpha}+\sqrt{3}\sin\theta^{\beta}+\cos\theta^{\beta}+1\right)^2+\left(3\delta-2\sqrt{3}\sin\theta^{\alpha}+\sqrt{3}\sin\theta^{\beta}-3\cos\theta^{\beta}+3\right)^2\right)}{3\sqrt{3}\mathrm{a}^2(\delta+1)^2}\\ \approx&\frac{8\left(\delta^2+\sqrt{3}\delta(\theta^{\beta}-\theta^{\alpha})+(\theta^{\alpha})^2-\theta^{\alpha}\theta^{\beta}+\theta^{\beta\,2}\right)}{\sqrt{3}\mathrm{a}^2(\delta+1)^2}\end{aligned}$$

Angles of the critical points (Divergence of A and zero n)

$$\theta^{\alpha} = -\theta^{\beta} = \tan^{-1}\left[\frac{-\sqrt{-(\delta-1)(\delta+3)}\delta-\sqrt{-(\delta-1)(\delta+3)}+\sqrt{3}}{\delta(\delta+2)-2}\right] \approx \frac{\delta}{\sqrt{3}}$$

More examples of double alignment

Following from our analysis in the main text, we can extend the comparison between the calculated periodicities to the electronic transport characteristics of three more devices. Figure S1 shows longitudinal resistance R_{xx}, hall resistance R_{xy} and their corresponding theoretical fittings of four samples; the device of the main text (fig. S1a), and three others (fig. S1b-d). All of these samples are encapsulated graphene heterostructures with perfect alignment in one side graphene-hBN contact surface. From the perfectly aligned moiré period, we can fit δ. The fitted parameters are shown in table S1.

We compare R_{xx} and R_{xy} data to confirm the presence of secondary Dirac points, whose signature has both a peak in the R_{xx} data and a reversal of sign in R_{xy}. The coexistence of these features rules out other features in the moiré minibands (*16*).

Table S1. δ and θ^{β} for each device. Lattice mismatch, δ, and angle of the second hBN, θ^{β}, for each device in fig. S1.

	Figure label	Lattice mismatch	Second twist angle
Sample 1	**a**	1.64%	0.4^0
Sample 2	**b**	1.66%	0.44^0
Sample 3	**c**	1.69%	1.25^0
Sample 4	**d**	1.71%	0.25^0

Many R_{xx} peaks are observable with good agreement with the calculated periods. The amplitudes of the R_{xx} peaks for super-moiré are typically smaller than those of the moiré Dirac points. This is particularly pronounced on the hole side where the amplitude ratios, shown in fig. S4, result in a big difference between peaks of moiré and super-moiré. The R_{xx} features match with features in R_{xy}, indicating the presence of a new Dirac point. Many of the super-moiré features in R_{xy} do not reverse the sign entirely – however this may be explained as a result of the weakened amplitude of the super-moiré scattering process.

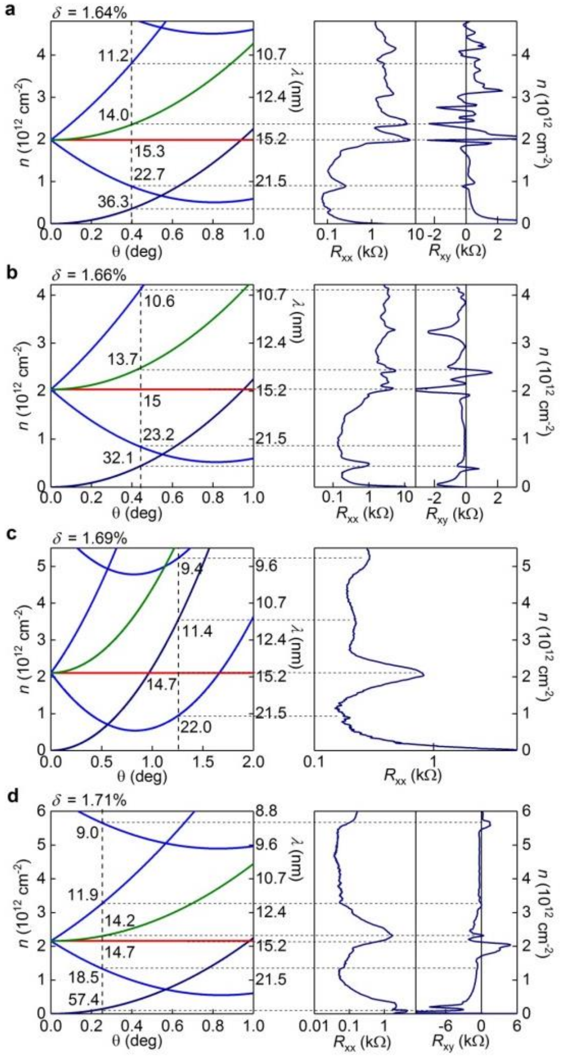

Fig. S1. Transport properties of double-aligned encapsulated graphene devices. a shows the calculated dependence on θ^{β} of the moiré and super-moiré periodicities. Dashed lines are a guide for the eye linking intersections with $\theta^{\beta} = 0.4$ to the R_{xx} and R_{xy} data. This is a reproduction of Fig. 3b and 3c from the main text. **b** Same as **a** for device two, $\theta^{\beta} = 0.44$. **c** same as **a** for device three, $\theta^{\beta} = 1.25$. **d** same as **a** for device 4, $\theta^{\beta} = 0.25$.

Different fundamental frequencies of Brown-Zak oscillations

Previous literature reported the Brown-Zak oscillations (BZO) as a robust method to study the periodic potential applied on the graphene, for which maxima in σ_{xx} occurs following $\frac{B}{B_F} = \frac{p}{q}$ where fundamental frequency $B_F = \frac{\phi_0}{S}$, p , q are integers, *S* is the area of unit cell (*27, 30*). Here we employ BZO to probe the unit cell resulting from moiré and super-moiré periodicities. The key point is to extract the different frequencies corresponding to different unit cells, and then calculate the periodicity by assuming it corresponds to a hexagonal area.

As reported in (*30*), with p, q increasing, the amplitude of BZO decays exponentially due to the smaller group velocity and larger super unit cell. Usually p = 1 is much more prominent than p = 2, 3... It is experimentally difficult to realise observations of the p=1, q=1, oscillations in singly-aligned graphene on hBN since the fundamental frequency is ~24T. Further, the BZO would be more visible at high carrier densities, high T (up to 150K), and in an electron doped graphene. The increased observation of the BZO at high temperature is due to the smearing of the electronic energy distribution which removes the influence of quantum phenomenon (Landau quantization). The BZO survive because they are transport oscillations, rather than a quantum oscillation.

In our BZO maps (fig. S2, fig. S3), the oscillation corresponding to an aligned graphene-hBN moiré is clearest. It is unobservable at carrier densities around Main Dirac points (MDP). However, other frequencies start to appear around the carrier densities of their corresponding R_{xx} peaks. Also, many BZO may be distinguished more clearly at high densities on electron-doped side of the main Dirac point (n>4.5e12 cm^{-2}) where the landau fan from the various Dirac point is unobservable. In fig. S2b, the oscillations of the second largest super-moiré start to appear around its R_{XX} peak 0.9e12cm^{-2} (fig. S1.**a**). In this range, only one frequency occurs at small magnetic fields, so it can be easily extracted. Then, around n = 2.0e12 cm^{-2}, shown in fig. S2.**c**, the frequency of perfectly aligned graphene-hBN moiré pattern becomes dominant. To distinguish other frequencies, higher n and higher B are both required. In fig. S2.d, we neglect the two frequencies that have already been extracted in the small n. Another two frequencies are observed. In total four distinct periodicities are observable; 2 moirés, and 2 super-moirés.

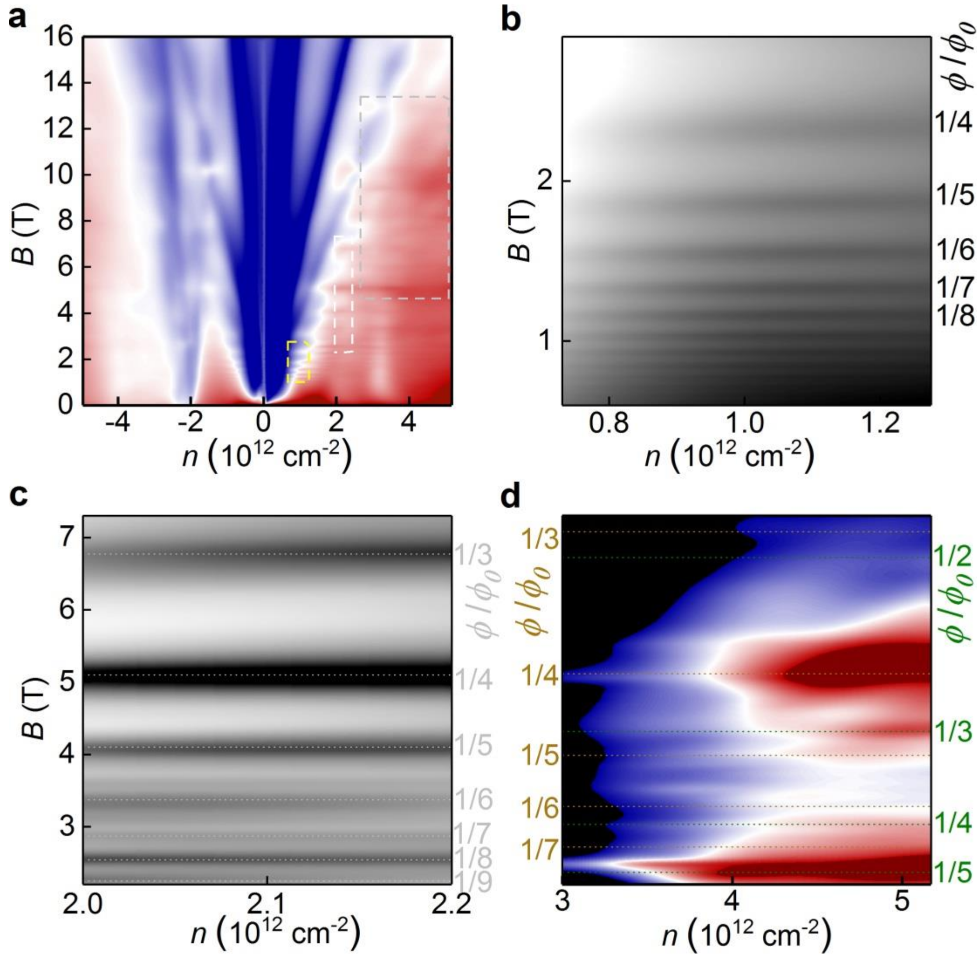

Fig. S2. Brown-Zak oscillations in sample 1. This sample is the same the main text. **a** σ_{xx} as a function of carrier concentration and magnetic field (Scale: blue to red 0.5 e^2/h to 70 e^2/h). **b**, **c** and **d** are zoom-in of the region marked by the yellow, white, and grey dashed lines, respectively (scales: blue to red, 7 e^2/h to 37 e^2/h.).

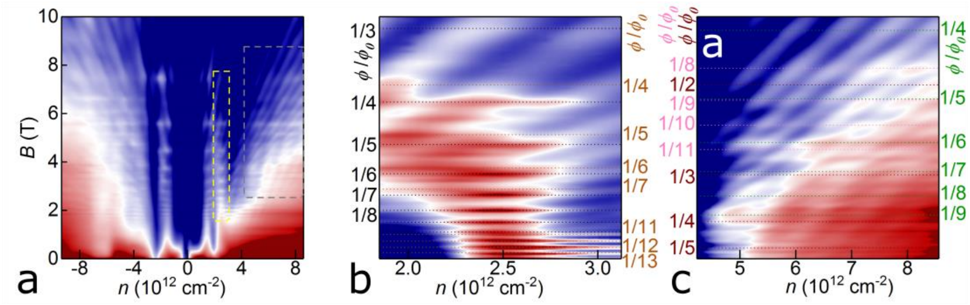

Fig. S3. Brown-Zak oscillations for sample 4. a σ_{xx} map of carrier concentration and B-field of sample (scale: blue to red, 4 e^2/h to 150 e^2/h). **b** and **c** are zoom-in of the **a**, marked by the yellow and grey rectangles, respectively. (**b** scale: blue to red, 2.4 e^2/h to 30 e^2/h) (**c** scale: blue to red, 3.5 e^2/h to 50 e^2/h). **d** shows two magnetic field sweepings at carrier densities 6.0e12cm^{-2} and 8.5e12cm^{-2} with the same scale as **c**, dotted lines connecting the peaks of two sweepings to the maxima in the map. The Brown-Zak oscillations correspond to a moiré structures of different periodicities are marked by dotted lines of different colours. Black – 14.6nm (B_F=22.5T), violet – 14.2nm (B_F=23.8T), light pink – 8.9nm (B_F =60.5T), green – 11.9nm (B_F=33.5T), red – 18.4nm (B_F=14.1T). The periods are similar with those derived by R_{xx} and R_{xy}. All measurements are done at 35K.

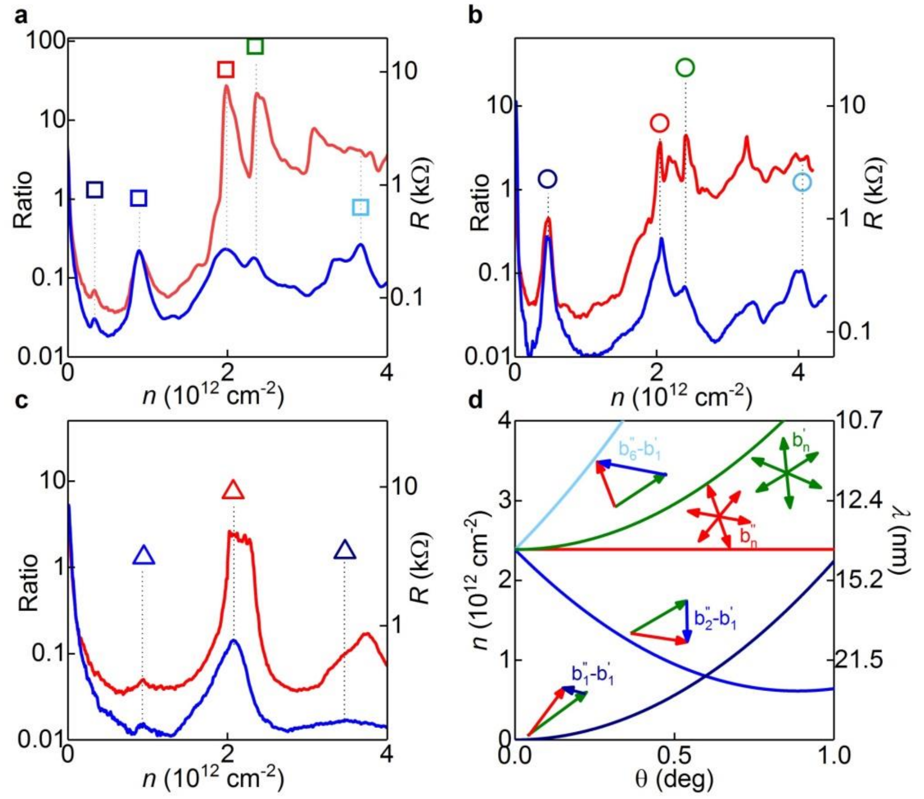

Fig. S4. Electron-hole symmetry in super-moiré features. a, b and **c,** scatter plots represent the ratios between hole-side peak amplitudes and their electron-side counterparts with dashed lines connecting their corresponding peaks (red – hole-side, blue – electron-side). Different shapes point to different samples, **a** (square) – sample 1, **b** (circle) – sample 2 and **c** (triangle) – sample 3, same as Main text Fig. 4. Different colours point to different moiré vectors and super-moiré vectors, shown clearly in **d**.

Gap opening at the main Dirac point

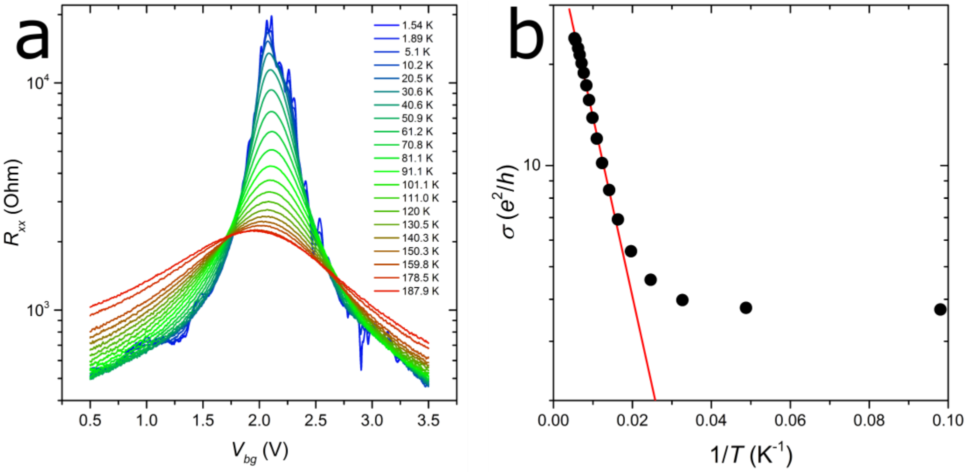

Fig. S5. Gap opening in one of our double-aligned samples. a R_{xx} vs back-gate voltage through the main Dirac point for various temperatures (1.54K-blue to 187.9K-red). **b** Temperature dependent conductivity at the main Dirac point. Experimental data (black dots) and exponential fit (red line). Fit gives a pseudo-gap of 21meV for data range of T>50K.

Previously, a gap has been observed in the singly-aligned graphene-hBN superlattices of approximately 25 meV (*18, 19*). The origin of the gap is in the proximity induced sublattice symmetry breaking in graphene due to the hBN layer. Hunt el Al (*18*), showed that the gaps size is correlated with the periodicity of the moiré pattern.

With each sample we have measured the temperature dependent behaviour of the conductivity at the main Dirac point. In fig. S5a we present the characteristic plot of R_{xx} against gate voltage through the main Dirac point, for many temperatures (1.54K – 187.9K). In fig. S5b, the σ_{xx} at the Dirac point is plotted against temperature. The gap may then be extracted by fitting the data with to the Arrhenius law. In this case, we observed a 21meV gap. Which is consistent with singly-aligned graphene on hBN.

AFM of other double-aligned samples

We have made and measured many doubly-aligned samples. Each sample shows a double moiré pattern in the AFM images (fig. S6 a-d), principally confirmed by the presence of two sets of independent spots in the Fourier transformation (fig. S6 e-h). In each case the encapsulation hBN layer is thin (<5 layers). We do not observe the moiré pattern when the hBN is thick (10s of nm). The double alignment is further confirmed by electronic transport data – yielding several moiré and super-moiré features.

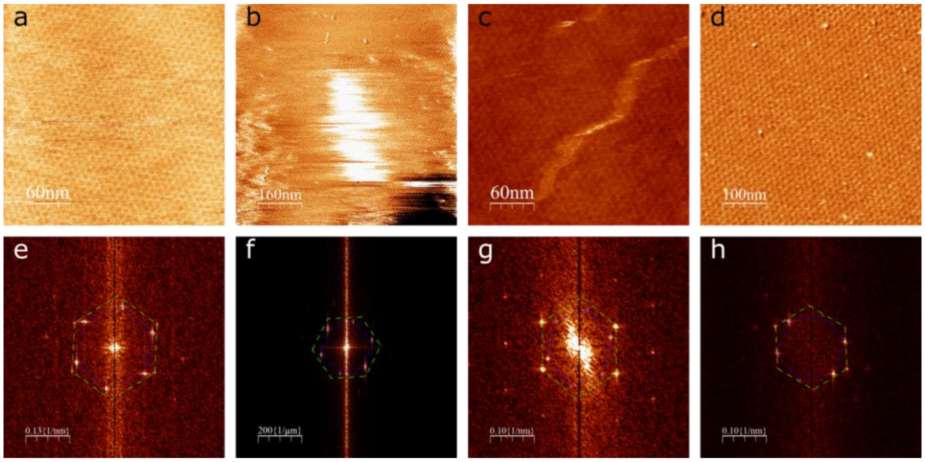

Fig. S6. Examples of double-aligned samples. a, b, c, and d AFM Young's Modulus images of super-moiré samples. In some cases, contamination is visible in the image. **e, f, g, and h** Fourier transformations of the images in **a, b, c, and d**, respectively. Each image shows two sets of hexagonal spots corresponding to distinct moiré periods.

Uniformity in heterostructures

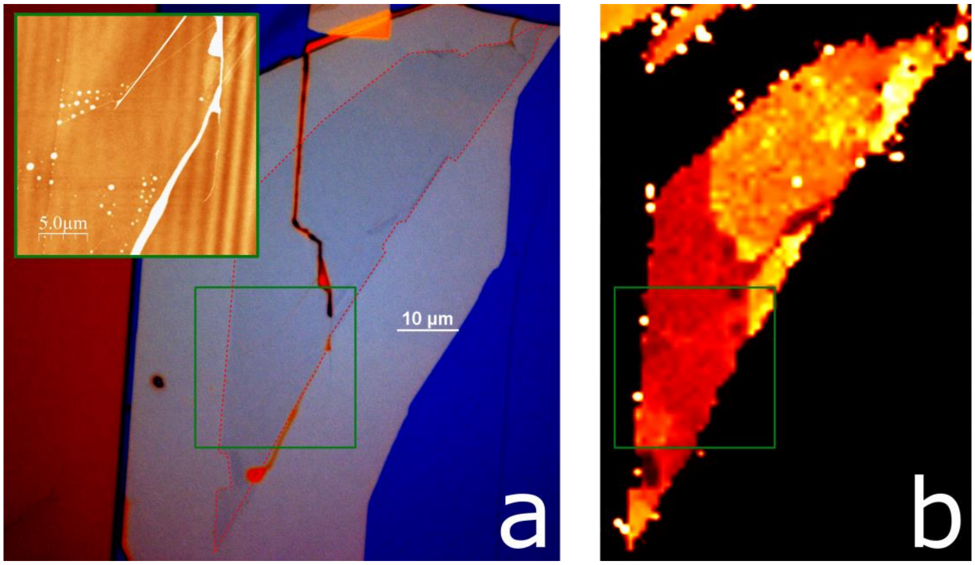

Fig. S7. Uniformity in double-aligned heterostructures. a Optical image (colour enhanced) of one of our hBN-graphene-hBN doubly aligned heterostructures before lithography. Dark blue highlights the bottom hBN layer, grey highlights overlap of the top hBN layer on the bottom hBN, and darker grey (outlined with dashed red) highlights the graphene region. **a-inset** AFM topography image of the area outlined by green in **a** and **b**. The vertical lines are an AFM artefact due to stray laser interference. Scale black to white is 2nm. **b** Map of the FWHM(2D) in the Raman spectrum. scale black to white is 15cm^{-1} to 100cm^{-1}. Each of these characterisation tools builds up an image of where there are distortions and deformations in the heterostructure. The lithography steps are then designed specifically to avoid these imperfections.

Typical heterostructures produced from mechanically exfoliated flakes (method described in the next section) contain imperfections (creases, folds, contamination blisters, tears, etc.). However, to confirm the uniformity of our doubly-aligned samples we utilise optical images, AFM, and Raman spectroscopy mapping, to characterise the nature of our fully assembled heterostructures (fig. S7) and design the devices to avoid such inhomogeneities. Figure S7a shows the optical images of one of our doubly aligned samples. Clearly evident is a crack in the top-hBN layer induced during the fabrication process. Likewise, the crack is visible in the FWHM(2D) map (fig. S7b). Further, in fig. S7a-inset we can see examples

of blisters and folds, which are most likely in the graphene layer. However, by only using the area of the heterostructures which is removed from these distortions we can guarantee uniform and homogenous devices.

Analysis of super-moiré peaks

The analysis of moiré patterns in hBN-GR-hBN was done following an approach described in Ref. (*33*). The methodology is based on the Fourier analysis of moiré patterns appearing when two hexagonal lattices are combined. A hexagonal lattice can be described using the function

$$f(\vec{r}) = \frac{1}{9} + \frac{8}{9}\cos\left(\frac{1}{2}\vec{k}_1\vec{r}\right)\cos\left(\frac{1}{2}\vec{k}_2\vec{r}\right)\cos\left(\frac{1}{2}\vec{k}_3\vec{r}\right) \qquad (25)$$

where $\vec{k}_1 = k[1,0]$, $\vec{k}_2 = k[-1,1]$, $\vec{k}_3 = k[0,-1]$, with $k = 4\frac{4\pi}{a\sqrt{3}}$, and a is the lattice constant. This function contains spatial frequencies at 0 (zero order), and $\vec{k}_1$, $\vec{k}_2$, $\vec{k}_3$ (first order). Higher order frequencies can be obtained when considering functions f^2 (second order) and f^3 (third order), or even f^n (n-th order). It is important to note that a perfect lattice would contain harmonics of infinite order with an amplitude in the Fourier transform that's inversely proportional to the order of the harmonic. Using the description of a lattice from Eq. (25), the moiré pattern defined by combining two of such lattices can be expressed as a product between functions f_1 and f_2. The Fourier transform of this product would reveal which spatial frequencies appear, and subsequently which are the possible moiré patterns.

We approach the problem of the trilayer heterostructures by considering two moiré patterns (one made by graphene and top hBN and the second one made by graphene and the bottom hBN) as the two lattices and following the approach explained above. Let’s define the moiré reciprocal vectors, $\vec{m}_{1,2}$ and $\vec{n}_{1,2}$ corresponding to graphene and hBN layers rotated by an angle θ_1 and θ_2, respectively

$$\vec{m}_i = \vec{g}_i \begin{bmatrix} \cos\theta_1 & -\sin\theta_1 \\ \sin\theta_1 & \cos\theta_1 \end{bmatrix} \frac{\vec{g}_i}{(1+\delta_1)} \quad (26)$$

and

$$\vec{n}_i = \vec{g}_i \begin{bmatrix} \cos\theta_2 & -\sin\theta_2 \\ \sin\theta_2 & \cos\theta_2 \end{bmatrix} \frac{\vec{g}_i}{(1+\delta_2)} \quad (27)$$

where $i = 1,2$, $\vec{g}_i$ are the reciprocal vectors of the graphene lattice, and δ_i are the relative ratios of unit cell lengths between graphene and the two hBN lattices. The super-moiré reciprocal vectors that correspond to different spatial periodicities can be defined as a difference between harmonics of different order (any two combinations of the underlying moiré vectors $\vec{m}_i$ and $\vec{n}_i$). Unlike the moiré pattern between graphene and hBN, the three layer problem (hBN-GR-hBN) (within a certain range of small rotation angles) results in higher order beating frequencies in the low frequency range, suggesting the emergence of structures with larger periodicity than the main pattern defined by the difference $\vec{m}_1$ - $\vec{n}_1$ (or $\vec{m}_2$ - $\vec{n}_2$). Figure S9 shows an example of frequency spectra obtained for $\theta_1 = 0^0$and $\theta_2 = 0.8^0$.

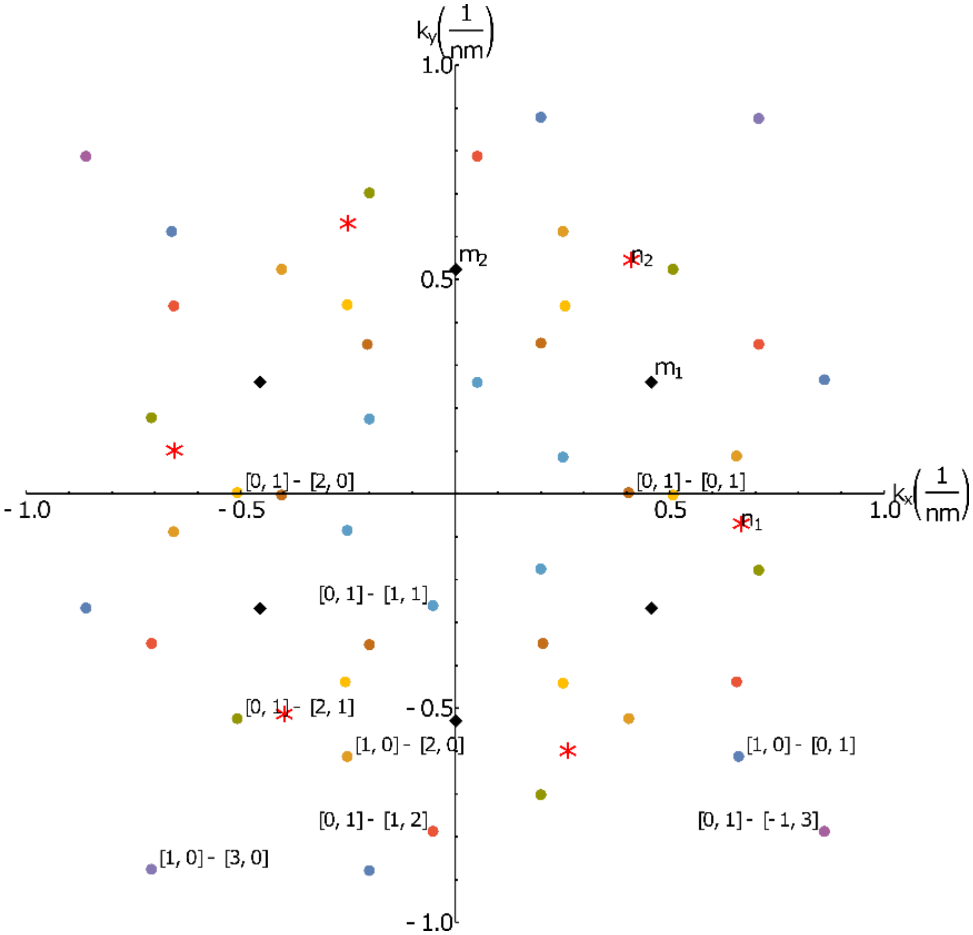

Fig. S8. Frequency analysis of different harmonics of hBN-gr-hBN structure. The reciprocal vectors of moiré patterns are marked with $m_{1,2}$ using $\theta_1 = 0^0$ (diamonds) and $n_{1,2}$using $\theta_2 = 0.8^0$ (stars) while the super-moiré harmonics are labelled as a difference between relative coordinates [p, q]−[r, s].

Dominant super-moiré harmonics that fit the experimentally observed features are labelled in fig. S10. They emerge as the difference between the moiré harmonics of different orders, as shown in the previous plot. Here, we show the super-moiré periods calculated using the following relation

$$\lambda = \frac{4\pi}{\sqrt{3}|\vec{k_i}|} \quad (4)$$

where $\vec{k}_i = (p\vec{m}_1 + q\vec{m}_2) - (r\vec{n}_1 + s\vec{n}_2)$, wh0ere p, q, r, s are integers.

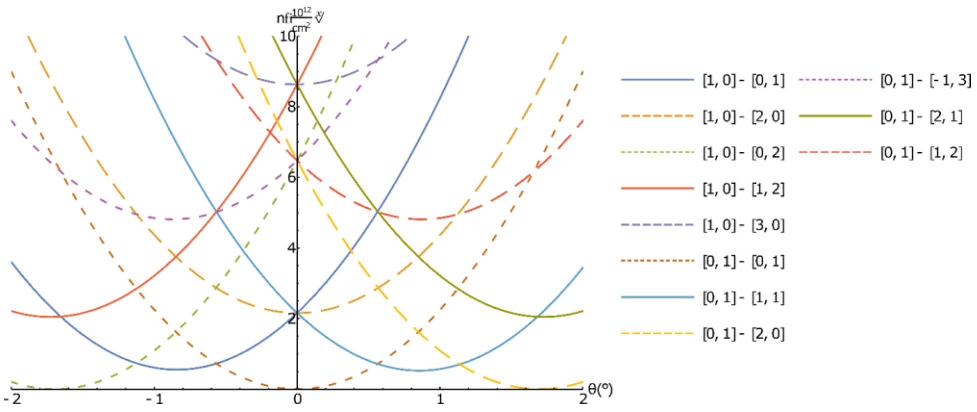

Fig. S9. Super-moiré periods corresponding to different harmonics. Curves obtained using different combination of [p, q]−[r, s] shown in the inset.

Tight-binding model

Tight-binding calculations are performed using standard tight-binding Hamiltonian given by

$$H = -\sum_{i,j} t(\vec{r}_i, \vec{r}_j) c_i^\dagger c_j \quad (28)$$

with

$$t(\vec{r}_i, \vec{r}_j) = V_\pi \left[1 - \left(\frac{\vec{d}\vec{e}_z}{d}\right)^2\right] + V_\sigma \left(\frac{\vec{d}\vec{e}_z}{d}\right)^2 \quad (29)$$

using

$$V_\pi = V_\pi^0 e^{-\frac{d-a_0}{\delta}}$$

$$V_\sigma = V_\sigma^0 e^{-\frac{d-d_0}{\delta}}$$

where $V_\pi^0 = -2.7$ eV, $V_\sigma^0 = 0.48$ eV are the intralayer and interlayer hopping integrals, respectively. $\vec{d}$ is the bond vector between sites i, j, a_0 is the equilibrium bond length of each material, and $\delta = 0.3187a_0$ is chosen to fit the next-nearest intralayer hopping value. Onsite terms of boron and nitrogen species are set to $V_B = 3.34\, eV$ and $V_N = -1.40\, eV$ respectively. Hoppings within the layer are considered up to the second nearest neighbour while the interlayer hoppings are considered within the radius of $1.5d_0$, where d_0 is the equilibrium interlayer distance. This radius is chosen due to the fact that hoppings outside this radius contribute only to a small energy shifts of the spectra, without modifying it significantly. However, neglecting the above mentioned hoppings results in a considerable gain in calculation time due to a reduced number of non-zero elements in the Hamiltonian.

Molecular dynamics simulations and Raman shift calculations

Molecular dynamics simulations are performed for the singly-aligned hBN/graphene and the double-aligned hBN/graphene/hBN by allowing the relaxations of both hBN and graphene layers. We used the bond-order Brenner potentials for the graphene layer, Tersoff potentials for the B-N interaction in the hBN layers and the Morse potential developed in Ref. (*34*) for the inter-layer interactions. The simulations are performed within the "large-scale atomic/molecular massively parallel simulator" (LAMMPS) (*35, 36*) by considering a disk of radius 120nm. We fix the atoms in a boundary region of 2nm but allow the relaxation of all other atoms. The total energy is minimized until the forces are below 10^{-6} eV/Å. Results of simulations are shown in fig. S11 where we plotted bond lengths of the relaxed graphene layer in the case of single and double aligned system.

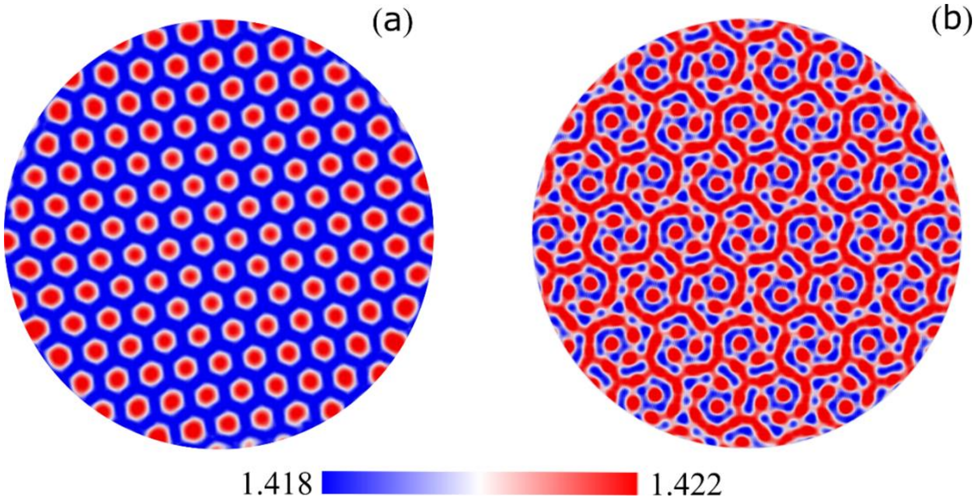

Fig. S10. Molecular dynamics simulations of bond lengths in graphene-hBN superlattices. Bond lengths in (a) single ($\theta = 0.4°$) and (b) double ($\theta_1 = 0$ and $\theta_2 = 0.4°$) aligned hBN/graphene/hBN system.

Using the relaxed structures, we calculate the shift of the 2D peak. Calculations are performed using the prescriptions given in Ref. (*37-39*). First, we obtain the strain tensor $\varepsilon(\boldsymbol{r})$ which is employed to extract the shift of the 2D peak as

$$\Delta\omega_{2D} = -\omega_{2D}^{0}\gamma_{2D}\varepsilon_{h} \tag{30}$$

where ω_{2D}^{0} is the 2D frequency of the unstrained graphene, $\gamma_{2D} = 3.55$ [5] , and $\varepsilon_h = \varepsilon_{xx} + \varepsilon_{yy}$ is the hydrostatic component of the strain tensor. This method has been applied to three different systems: graphene aligned with both hBN layers, graphene aligned with top hBN layer, and non-aligned system (Fig. 5b red, blue, and black curve, respectively, in the main text). A histogram of the shifts has been produced and the Lorentzian fitting to the obtained data was performed.
